# Sub-national variation in measles vaccine coverage and outbreak risk: a case study from a 2010 outbreak in Malawi

**DOI:** 10.1186/s12889-018-5628-x

**Published:** 2018-06-15

**Authors:** Avery Kundrick, Zhuojie Huang, Spencer Carran, Matthew Kagoli, Rebecca Freeman Grais, Northan Hurtado, Matthew Ferrari

**Affiliations:** 1 Hershey Medical School, The Pennsylvania State University, Hershey, PA USA; 2 Department of Biology, The Pennsylvania State University, University Park, PA USA; 3 Ministry of Health, Lilongwe, Malawi; 4 Epicentre, Paris, France; 5 Medecins Sans Frontieres, New York, NY USA

**Keywords:** Measles, Outbreak, Equity, Vaccination

## Abstract

**Background:**

Despite progress towards increasing global vaccination coverage, measles continues to be one of the leading, preventable causes of death among children worldwide. Whether and how to target sub-national areas for vaccination campaigns continues to remain a question. We analyzed three metrics for prioritizing target areas: vaccination coverage, susceptible birth cohort, and the effective reproductive ratio (R_E_) in the context of the 2010 measles epidemic in Malawi.

**Methods:**

Using case-based surveillance data from the 2010 measles outbreak in Malawi, we estimated vaccination coverage from the proportion of cases reporting with a history of prior vaccination at the district and health facility catchment scale. Health facility catchments were defined as the set of locations closer to a given health facility than to any other. We combined these estimates with regional birth rates to estimate the size of the annual susceptible birth cohort. We also estimated the effective reproductive ratio, R_E_, at the health facility polygon scale based on the observed rate of exponential increase of the epidemic. We combined these estimates to identify spatial regions that would be of high priority for supplemental vaccination activities.

**Results:**

The estimated vaccination coverage across all districts was 84%, but ranged from 61 to 99%. We found that 8 districts and 354 health facility catchments had estimated vaccination coverage below 80%. Areas that had highest birth cohort size were frequently large urban centers that had high vaccination coverage. The estimated R_E_ ranged between 1 and 2.56. The ranking of districts and health facility catchments as priority areas varied depending on the measure used.

**Conclusions:**

Each metric for prioritization may result in discrete target areas for vaccination campaigns; thus, there are tradeoffs to choosing one metric over another. However, in some cases, certain areas may be prioritized by all three metrics. These areas should be treated with particular concern. Furthermore, the spatial scale at which each metric is calculated impacts the resulting prioritization and should also be considered when prioritizing areas for vaccination campaigns. These methods may be used to allocate effort for prophylactic campaigns or to prioritize response for outbreak response vaccination.

## Background

Despite progress towards increasing global measles vaccination coverage and decreasing measles-related mortality, measles continues to be one of the leading, preventable causes of death among young children worldwide [1]. Approximately 145,700 people – primarily children under the age of 5 – died from measles in 2013 [1] and large measles outbreaks still occur in five of the six World Health Organization regions [1–6]. Large resurgent outbreaks in southern Africa in 2010–11 [2] and in 2015 in Democratic Republic of Congo [7] have highlighted the fragility of measles elimination efforts, even in settings that have experienced large periods of low incidence. These outbreaks have highlighted gaps in current measles vaccination programs and emphasize the need to evaluate current vaccination and consider the development of new strategies [8].

Effective measles control requires strategies for increasing prophylactic vaccination and reacting to outbreaks when they occur [9]. These strategies must be tailored to each country’s specific needs: local variation in access to routine vaccination, history of supplemental vaccination campaigns, and epidemic history can generate significant sub-national variation in the distribution of immunization and susceptible children [10]. These local heterogeneities may contribute to regional persistence as poorly immunized areas serve as reservoirs of transmission or “hotspots” for epidemic invasion [11]. Further, local heterogeneity in vaccination coverage, the number of children requiring vaccination, and the accessibility of local populations can limit the effectiveness of supplemental and outbreak response vaccination activities [8]. Policies to reduce measles transmission at the national or regional scale must balance the competing objectives of equitable distribution of vaccination resources and minimizing outbreak and transmission risk [8, 12]. How to most effectively prioritize locations for vaccination campaigns before or during an epidemic remains an open question [8].

Vaccination coverage serves as a primary indicator of measles control [13]. The World Health Organization’s stated goal is to achieve 90% vaccination coverage with the first routine dose of a measles-containing vaccine and to exceed 80% vaccination coverage in every district or equivalent by the end of 2015 [9]. This recommendation highlights two separate objectives: 1) obtaining high national vaccination coverage and 2) obtaining equitable sub-national vaccination coverage [9].

Local scale measures of vaccination program performance are scarce. Vaccination coverage is typically calculated by administrative methods – comparing the number of vaccine doses to the target population. This method does not account for vaccination of individuals outside of the targeted area, re-vaccination of individuals already vaccinated, and the inaccessibility of some sub-populations [14], and often does not account for vaccine wastage (i.e. discarded doses). Population estimates are often not up to date and produce inflated administrative estimates in comparison to population-based surveys [13]. Not all vaccine doses lead to a new immunized individual (e.g. if the individual was previously immunized); therefore, vaccination coverage values do not directly describe population immunity [14].

The International Society for Equity in Health, defines equitability as “the absence of systematic and potentially remediable differences in one or more aspects of health across population sub-groups defined socially, economically, demographically, or geographically” [15]. Achieving equitable vaccination coverage implies a need to target low coverage areas, which may also serve as “hotspots” for epidemic transmission. National administrative vaccine coverage levels may mask discrepancies in impoverished or isolated populations [10, 15]. Vaccination coverage at the local scale better reflects the susceptibility of populations to disease and could be used to prioritize vaccination targets before and during an outbreak [10, 15].

Vaccination coverage alone does not account for the absolute number of susceptible individuals. Ferrari et al. [13] showed that the persistence of measles at the national-scale correlates with the size of the annual birth cohort (i.e. children born who will not be vaccinated) more so than vaccination coverage alone [13]. Large populations, or populations with high birth rates, may disproportionately contribute to the annual cohort of susceptible children than do small populations, even if the latter has lower vaccination coverage. Considering both local vaccination coverage and susceptible birth cohort may be more effective in prioritizing vaccination campaign targets than vaccination coverage alone [13].

The effective reproductive ratio (R_E_), the average number of secondary cases that result from a single infectious individual in a partially immunized population (i.e. those with a combination of natural and vaccine derived immunity), is a classic measure of outbreak risk [16]. R_E_ varies within populations as a function of the proportion immune [17], birth rates [16], population density and contact rates [16, 18], and mixing between age classes [19]. Large-scale population estimates which do not account for the heterogeneity of R_E_ conceal clusters of susceptible individuals that may increase the susceptibility of the larger population to an outbreak [11, 20, 21].

After several years of declining measles incidence, Malawi experienced a resurgent outbreak with over 130,000 reported cases [2] in 2010. In response to this outbreak, several vaccination campaigns were conducted, first by the Malawi Ministry of Health (MoH) and then with additional support from Medecins Sans Frontieres. Enhanced surveillance during the outbreak response (see Methods) resulted in a highly spatially resolved line list of reported measles cases during this outbreak. Here, we quantify the local variation in estimated vaccination coverage, the annual susceptible birth cohort, and R_E_ at the district and the individual health facility scale in Malawi based on data collected during 2010. The outbreak revealed significant sub-national spatial heterogeneity in these measures, which suggests the potential relevance of locally-specific strategies to achieve an equitable distribution of risk. Thus, this the Malawi outbreak provides a retrospective case-study through which we can quantify spatial heterogeneity and evaluate the potential for the development of future outbreak response policies that are reactive to the local epidemiological context [2, 8]. We make recommendations for prioritizing regions with respect to separate goals of 1) achieving equitable high vaccination coverage and 2) minimizing the size of the susceptible birth cohort and reducing R_E_. These measures need not result in the same prioritization of target areas; however we highlight the overlapping target areas that are prioritized by multiple measures.

## Methods

Through retrospective review of health registers and weekly communication to the district level, Epicentre and the Malawi MoH created a line list of all measles cases presented at health facilities [2]. A positive measles case was recorded if the patient was exhibiting a generalized maculopapular rash, a fever of ≥38 °C, and at least one of the following: cough, runny nose, or conjunctivitis or if the patient was diagnosed with measles by a health professional [2]. For each suspected measles case, the date of onset, date of clinic visit, epidemic week of clinic visit, location and name of health facility, age, and vaccine history (recorded as positive if the patient had a vaccine card or if the patients mother reported positive vaccination status) [2]. The complete line list contained 129,037 entries.

We obtained fertility (births/female) at a regional scale (North, Central, and South) from the 2008 Malawi Census.

We recorded the date of consultation at a health facility, the health facility name, and the epidemic week for each case in the line list. Where possible, the date of consultation was verified against the corresponding epidemic week and the health facility name was verified against maps of known health facilities provided by the Malawi Ministry of Health and the National Statistical Office of Malawi. Records for which the date (5676 records) or health facility (22,925 records) could not be verified were discarded. After correction, the line list included 100,436 entries of the original 129,037.

Using the combined reference map lists of health facilities from the Malawi Ministry of Health and the National Statistical Office of Malawi (above), we mapped the point location all of the health facilities. We approximated the catchment areas around each health facility using a Voronoi tessellation; the resulting region around each health facility, which we refer to hereafter as a health facility polygon, contains the set of all points nearer to a given health facility than to any other health facility. We generated a GIS shapefile with all health facility polygons. Of the 1092 health facility polygons, 390 had reported cases and, of those, 338 had recorded the vaccination history of the patient. Population sizes for each polygon were derived from the WorldPop project (www.worldpop.org.uk).

We estimated vaccination coverage, VC, using the relationship proposed by Orenstein et al. [22],

1 $$ \mathrm{VC}=\frac{\mathrm{PCV}}{\left(1-\mathrm{VE}+\mathrm{PCV}\times \mathrm{VE}\right)} $$

where (PCV) is the proportion of measles cases with a history of vaccination and VE is the measles vaccine efficacy. We assumed a vaccine efficacy of 0.85 [23]. We estimated VC at both the district scale and health facility polygon scale.

The number of measles cases and the proportion of individuals with recorded vaccination history were highly variable among health facilities. To generate an interpretable surface of vaccine coverage, we first performed a spatial smooth of the health facility level PCV values. We calculated smoothed PCV for each polygon by dividing the sum of all the cases with positive vaccination history reporting to health facilities within a radius of 10 Km of the reference polygon by the sum of all the cases with vaccination history within the same radius. We applied eq. 1 to estimate smoothed VC values using the smoothed PCV values. This allowed estimation of vaccine coverage for health facility polygons with no reported data on the vaccination history of cases.

We estimated the annual number of children born, who will not be vaccinated, Y_j_, as:

$$ {Y}_j={\mathrm{N}}_j\times {\mathrm{F}}_j\times \left(1-{\mathrm{VC}}_j\right) $$

where N_J_ is the female population size for that spatial unit, j, F_j_ is the number of live births per female in the region (North, Central, and South) that contains spatial unit j, and VC_j_ is the estimated vaccination coverage for that spatial unit j. Hereafter, we refer to Y_j_ as the susceptible birth cohort.

To estimate the susceptible birth cohort at the district scale, we used female population sizes from the 2008 Census. At the health facility polygon scale, we assumed the female population to be 50% of the population which was derived from WorldPop estimates. Fertility was assumed to be the regional fertility at both spatial scales.

We estimated the effective reproductive ratio (R_E_) for each health facility polygon using the estimator given in [24],

$$ {\mathrm{R}}_{\mathrm{E}}=1+\mathrm{IG}+\mathrm{F}\left(1-\mathrm{F}\right){\left(\mathrm{IG}\right)}^2 $$

where I is the mean serial interval, G is the exponential growth rate of the cumulative number of cases, and F is the ratio of the infectious period to the serial interval. We assumed a 14-day serial interval for measles infection. G is given by

$$ \mathrm{G}\left(\mathrm{t}\right)=\frac{\ln \left({\mathrm{S}}_t\right)}{\mathrm{t}} $$

where S_t_ is the 33rd percentile of cases and t is the time it took for the first 33rd percentile of cases to present to health facilities within a polygon. We chose the first 33% of cases as this was early enough in the epidemic that dynamics were still in the exponential growth phase; consistent with the assumptions of [24]. F is assumed to be 0.5.

## Results

**Fig. 1:**
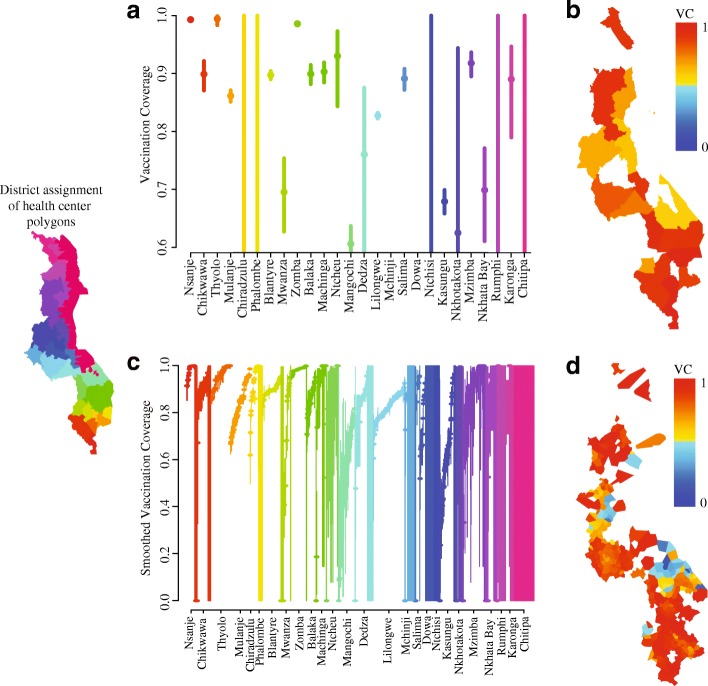
Estimated vaccination coverage (VC) at the district scale and health facility polygon scale. **a** Estimated VC for each district (solid circles), vertical lines indicate 95% confidence intervals. Intervals spanning 0-1indicate indeterminate estimates. Districts were ordered from south to north and colors correspond to the inset map. **b** Estimated VC at the district scale plotted on a scale from blue (VC = 0) to red (VC = 1.0). Areas with no information are indicated in white. **c** Estimated smoothed VC for each health facility polygon is indicated with a solid circle and vertical lines indicate 95% confidence intervals. Polygons are grouped by the district that contains their centroid and arranged by increasing VC. Districts are ordered from south to north and colors correspond to the inset map. **d** Estimated smoothed VC at the health facility polygon scale plotted on a scale from blue (VC = 0) to red (VC = 1.0). Areas with no information are indicated in white. Maps were generated by the authors. Province boundaries were extracted from a shape file provided by the Malawi MoH. Health facility boundaries were generated as described in the Methods

The mean estimated vaccination coverage across all districts was 84% (range: 61–99%; Fig. 1a and b). Six districts (Mangochi, Kasungu, Nkhata Bay, Mwanza, Nkhotakota, and Dedza) had estimated vaccination coverage below 80%. Mangochi, in central Malawi, had the lowest estimated vaccination coverage of 61% (CI: 57–64%).

Vaccination coverage at the health polygon level resembled coverage at the district level, but was more variable; coverage at the health polygon level ranged from 0 to 100% (Fig. 1c and d). The health polygons within the district of Mangochi had uniformly low coverage, which was consistent with the district level estimate. However, many other districts did not follow this pattern; Kasungu had district level coverage of 68% (CI: 66–70%), but the health polygon level estimates ranged from 0% (CI: 0–70.6%) to 94.8% (CI: 91.9–96.8%). Interestingly, in the district of Lilongwe in central Malawi, estimated vaccination coverage increased radially outward from the center of Lilongwe City.

**Fig. 2:**
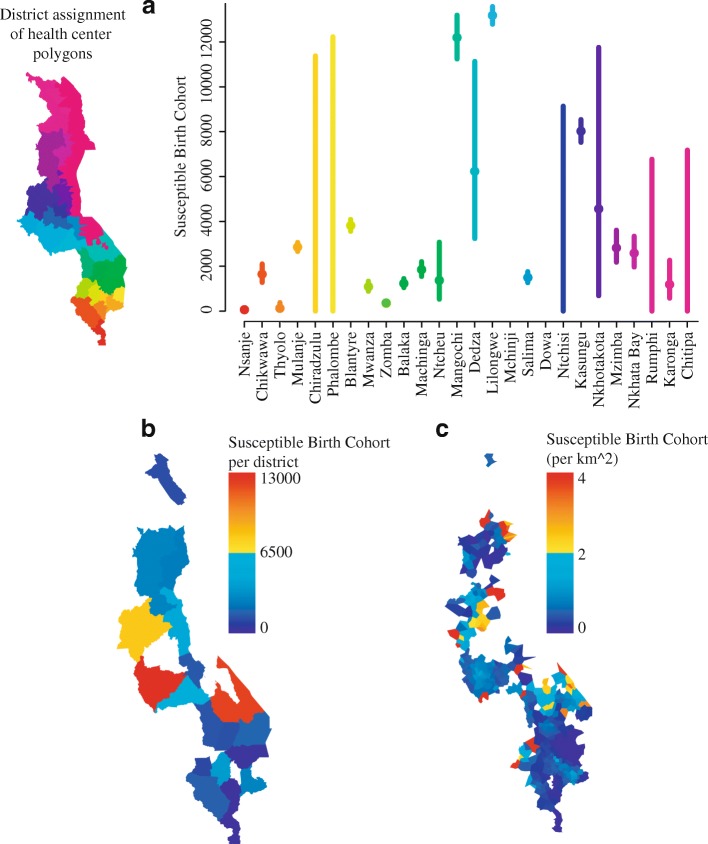
Estimated annual susceptible birth cohort at the district scale and health facility polygon scale. **a** The absolute size of the susceptible birth cohort at the district scale is indicated with a solid circle and vertical lines represent a 95% confidence interval. Districts were organized from south to north with colors corresponding to the reference map. **b** The size of the susceptible birth cohort is plotted on a scale from blue to red (range = 0, 13,000). Areas with no information are indicated in white. **c** The susceptible birth cohort per health facility polygon area plotted on a scale from blue to red (range = 0–4 per km^2^). Areas with no information are indicated in white. Maps were generated by the authors. Province boundaries were extracted from a shape file provided by the Malawi MoH. Health facility boundaries were generated as described in the Methods

The mean susceptible birth cohort per district was 3510, but the value varied greatly from district to district (64–13,182, Fig. 2a and b). The district of Mangochi, which had the lowest vaccination coverage, unsurprisingly had a susceptible birth cohort of 12,196 children per year (CI: 11,241–13,199). Lilongwe, which had relatively high vaccination coverage of 83% (CI: 82–83%), had the highest susceptible birth cohort of 13,182 (CI: 12,792–13,582). These results demonstrate that vaccine coverage alone may obscure the absolute contribution of each spatial unit to the regional susceptible pool.

The susceptible birth cohort at the health facility polygon scale reveals high local variability in the contribution to the regional susceptible pool (Fig. 2c). At the district scale, Lilongwe, Mangochi, and Kasungu had the largest annual contribution to the absolute number of susceptibles. Within the districts of Mangochi and Kasungu, the health facility polygons with the largest contribution to the annual susceptible birth cohort were highly clustered. In the district of Lilongwe, though vaccination coverage (Fig. 1d) tended to increase with distance from the city center, the higher population density in the center of the city suggests that the outlying health facility polygons contributed relatively fewer susceptibles on an annual basis (Fig. 2c).

**Fig. 3:**
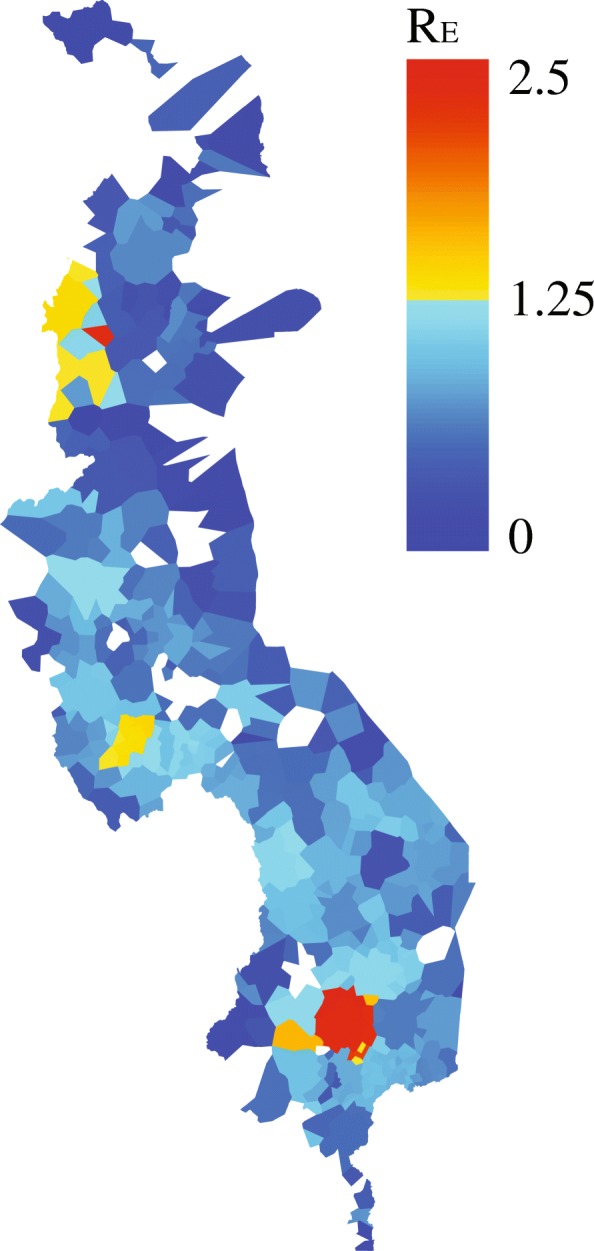
Estimates of RE at the health facility polygon scale. RE values were plotted on a scale from blue (RE = 0) to red (RE = 2.56). Areas with no information are indicated in white. Map was generated by the authors. Health facility boundaries were generated as described in the Methods

Across Malawi, R_E_ varied from 1.00 to 2.56 at the polygon scale. The effective reproductive ratio (R_E_) at the polygon scale was the highest in southern Malawi and lowest in northern Malawi with the exception of two clusters of health facility polygons with high R_E_ values in Mzimba and Lilongwe (Fig. 3). Health facility polygons within the district of Blantyre had the highest R_E_ values. We note that polygons in which no cases were reported may reflect areas where R_E_ was below the invasion threshold of 1 or areas that have little contact with their neighbors and therefore were not exposed to infection.

Each of the measures describe different characteristics of the measles epidemiology: VC is a measure of the vaccination program, the susceptible birth cohort is a measure of potential outbreak size, and R_E_ is a measure of both the speed of an outbreak and expected outbreak size [16]. Areas that rank as high risk with respect to multiple measures may be of particular concern and warrant high priority for supplemental vaccination.

**Fig. 4:**
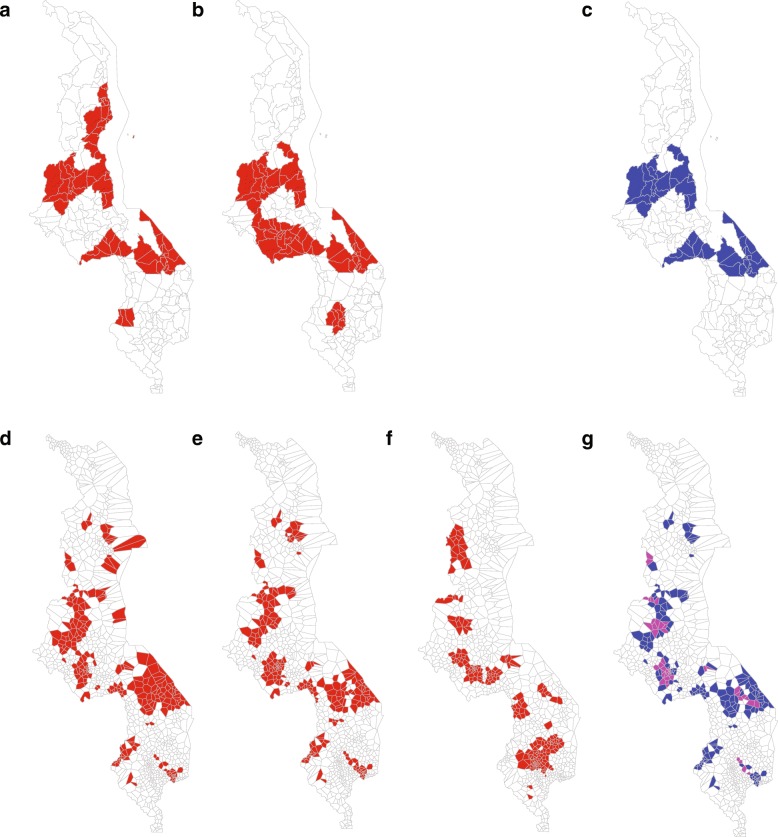
Highest priority targets at district (**a**–**c**) and health facility polygon scale (**d**–**g**). **a** Districts with vaccination coverage less than 80%. **b** The four districts with the largest susceptible birth cohort. **c** Districts prioritized by both VC and susceptible birth cohort at the district scale. **d** Health facility polygons with VC values less than 80%. **e** The 254 polygons with the largest susceptible birth cohort. **f** The 258 polygons with the highest values for R_E_. **g** Health facility polygons prioritized by two metrics (blue) or all three metrics (purple) as seen in **d**, **e**, and **f**. Maps were generated by the authors. Province boundaries were extracted from a shape file provided by the Malawi MoH. Health facility boundaries were generated as described in the Methods

At the district level, we found six districts (Mangochi, Kasungu, Nkhata Bay, Mwanza, Nkhotakota, and Dedza) with estimated vaccination coverage less than 80% (Fig. 4a). To make a similar comparison, we mapped the six districts (Mangochi, Dedza, Lilongwe, and, Kasungu, Nkhotakota, and Blantyre) with the largest susceptible birth cohort (Fig. 4b). Only 4 districts were among the 6 at highest risk with respect to both VC and susceptible birth cohort: Mangochi, Dedza, Kasungu, and Nkhotakota (Fig. 4c).

At the health facility polygon scale, we found 254 polygons with VC estimates less than 80% (Fig. 4d). To make parallel comparisons, we mapped the 254 “target” polygons with the highest annual susceptible birth cohort per polygon area (Fig. 4e) and the 258 (8 polygons had equal values) polygons with the highest values for R_E_ (Fig. 4f). The measurements of VC and susceptible birth cohort had similar spatial distribution, varying only slightly in the areas of Lilongwe and Mangochi. Mangochi had more target polygons when using VC to prioritize targets and Lilongwe had more target polygons when susceptible birth cohort was used to prioritize targets. Conversely, the distribution of R_E_ differed greatly from that of VC and susceptible birth cohort. The highest R_E_ values were in health facility polygons near Blantyre, Ntcheu, Lilongwe, and Mzimba whereas the highest values for VC and susceptible birth cohort were in the areas of Mangochi, Lilongwe, and Kasungu. The districts of Mangochi, Lilongwe, and Kasungu were prioritized by all three measurements at the health facility polygon scale (Fig. 4g).

## Discussion

The 2010 measles outbreak in Malawi highlighted that high vaccination coverage, while an important objective, is not necessarily sufficient to prevent outbreaks; the outbreak began and spread fastest in regions around Blantyre, where estimated vaccination coverage was high. This suggests a benefit of thinking more broadly about measures for evaluating vaccination program performance and prioritizing sub-national areas for epidemic surveillance and supplemental campaigns. We present three measures – vaccination coverage, susceptible birth cohort, and R_E_ – for assessing epidemic risk, and show that these measures provide alternative views of risk and prioritization for supplemental action.

We found that vaccination coverage and susceptible birth cohort estimates resulted in relatively similar prioritization of target areas, primarily in the regions of Mangochi and Kasungu, at both the district and health facility polygon scale. However, R_E_ estimates resulted in an alternative prioritization, notably in the region of Blantyre, which had relatively high vaccination coverage and low contribution to susceptible birth cohort. Additionally, we found that the scale at which each measurement was estimated had an impact on prioritization. District scale measurements masked significant heterogeneity at the health facility polygon scale.

Each metric – vaccination coverage, susceptible birth cohort, and R_E_ – reflects different epidemiological characteristics. Vaccination coverage and susceptible birth cohort measure the size of the population at risk, whereas R_E_ measures the rate of transmission and the proportion of at-risk individuals that will be affected by an outbreak [16]. Accordingly, vaccination coverage and susceptible birth cohort estimates resulted in relatively similar prioritization of target areas, which correspond to areas to which the 2010 Malawi outbreak spread, once established. Estimates of R_E_ resulted in the prioritization of locations within the district of Blantyre, which is urban and densely populated, and correspond to areas where the 2010 outbreak originated.

Consequently, there are tradeoffs to choosing one metric for prioritization over another. Targeting areas with low vaccination coverage and high susceptible birth cohorts may decrease the size of the population at risk and prevent further spread of an outbreak, whereas targeting areas with high R_E_ will decrease the risk of an outbreak starting. Despite these differences, some areas within the districts of Lilongwe, Kasungu, and Mangochi were prioritized by all three metrics; such regions may warrant particular attention. Our analyses demonstrate the need to consider multiple metrics for prioritization when making public health decisions.

We found that large-scale, district estimates masked substantial variation at the scale of health facility polygons. These results match previous findings and bolster the notion that small scale estimates of vaccination coverage, susceptible birth cohort, and R_E_ may better reflect the probability of an epidemic emerging within a population [10, 11, 15]. Operational response at small scales may allow better targeting of resources but must be balanced against logistical constraints.

A limitation of this study is that the analyses were completed retrospectively; therefore, the prioritization suggested here cannot be taken as a criticism of the 2010 outbreak response. However, if the information presented in this study had been available, prioritization of outbreak response immunization may have been differently allocated. We found that areas within Lilongwe, Kasungu, and Mangochi were prioritized by all three measures. Of the eight districts with vaccination campaigns, Lilongwe and Mangochi were fourth and fifth (week 24 of the epidemic) to receive campaigns and Kasungu received no vaccination campaigns [2]. The second and third districts to receive vaccination campaigns were Mzimba (week 19) and Chiradzulu (week 20) [2]; these districts were not prioritized by any of the three metrics we used. Blantyre, which was prioritized based on R_E_, was the first district to receive vaccination campaigns (week 18) [2]. Although our estimates were calculated retrospectively, estimating vaccination coverage and susceptible birth cohort is possible before or during an outbreak [14, 22, 25] and could be used to allocate resources during an outbreak, such as the recent epidemic in the Democratic Republic of Congo [8]. Estimating R_E_ before or during an outbreak is more difficult, generally requiring the use of models [17]. However, since we only used the first 33% of cases in our calculation, R_E_ could be estimated during the initial phase of an epidemic in a similar fashion [24].

While a retrospective analysis, such as this, is too late for to help during an outbreak, such post-outbreak analysis can highlight gaps in current health systems and high-risk areas that could be targeted for additional effort in routine or supplemental immunization. For example, the low proportion of cases with vaccination history in Mangochi (Fig. 1) suggests low routine coverage. This might suggest the need for further follow-up in low coverage areas to identify and close gaps in routine immunization services.

A further limitation of this study is that our estimates relied on surveillance data. Therefore, our estimates depend on the quality of the data recorded, the areas the outbreak reached, and the number of cases that occurred in each location. If the outbreak did not reach a particular location, no information was recorded for that area for us to use in our analyses.

## Conclusions

Although substantial progress towards the eradication of measles has been made worldwide, measles outbreaks continue to occur, suggesting the need to evaluate current measles control methods [8]. Logistical and accessibility constraints often limit the reach of both routine and supplemental vaccination services; sub-national evaluation of vaccination coverage, susceptible birth cohort, and outbreak risk provide an opportunity to adapt vaccine strategies to local needs [8]. The eradication of measles requires effective outbreak response procedures in addition to increasing vaccination coverage to obtain and maintain high levels of herd immunity. Routine immunization, supplemental immunization activities and outbreak response immunization are all critical tactics used to achieve this goal. However, because of limited resources, not all areas can be targeted immediately; thus, prioritization of target areas is necessary. Here, we have demonstrated that numerous metrics for prioritization exist and result in discrete prioritizations, that some areas are prioritized by multiple metrics, and that prioritizations vary based on the spatial scale. When considering which metric for prioritization to use, public health officials should consider multiple factors such as the country’s measles control objectives, the local demographics, and the epidemiology of the initial phase of the epidemic [26]. Prioritization of target areas should be context-specific in order to achieve optimal allocation of vaccination campaigns through a balance of epidemiological risk and logistical constraints [8, 26].

## Abbreviations

MoH: Ministry of Health
PCV: Proportion of measles cases with a history of vaccination
R_E_: Effective reproductive ratio
VC: Vaccine coverage
VE: Vaccine effectiveness
